# Efficacy of intravenous iron treatment for chemotherapy-induced anemia: A prospective Phase II pilot clinical trial in South Korea

**DOI:** 10.1371/journal.pmed.1003091

**Published:** 2020-06-08

**Authors:** Jun Ho Jang, Youjin Kim, Silvia Park, Kihyun Kim, Seok Jin Kim, Won Seog Kim, Chul Won Jung, Jeeyun Lee, Se-Hoon Lee

**Affiliations:** 1 Division of Hematology-Oncology, Department of Medicine, Samsung Medical Center, Sungkyunkwan University of School of Medicine, Seoul, South Korea; 2 Division of Hematology-Oncology, Department of Medicine, Samsung Changwon Hospital, Sungkyunkwan University School of Medicine, Changwon, South Korea; 3 Department of Hematology, Catholic Hematology Hospital, Seoul St. Mary’s Hospital, College of Medicine, The Catholic University of Korea, Seoul, South Korea; 4 Leukemia Research Institute, College of Medicine, The Catholic University of Korea, Seoul, South Korea; University of Pittsburgh, UNITED STATES

## Abstract

**Background:**

Anemia is the most common and serious cancer-related complication. This study aimed to evaluate the efficacy of administration of ferric carboxymaltose without erythropoiesis-stimulating agents for treating anemia in cancer patients. Moreover, we identified the biomarkers of hemoglobin response to predict the need for iron therapy.

**Methods and findings:**

We enrolled patients with solid cancers who were treated at a single institute (Samsung Medical Center, South Korea), from April 2015 to July 2017, in this prospective single-arm Phase II clinical trial. Patients received intravenous ferric carboxymaltose (1,000 mg) infusion on the first day (visit 1) of treatment. The primary end point was the number of hemoglobin responders, defined as patients with an increase in hemoglobin level ≥ 1.0 g/dL from the baseline, a hemoglobin level ≥ 11.0 g/dL, or both, within an 8-week observation period (week 3, 6, or 8). Secondary end points included changes in transferrin saturation and levels of soluble transferrin receptors, hepcidin, erythropoietin, interleukin-6, and C**-**reactive protein (CRP) at each visit. Of the 103 recruited patients, 92 were eligible for analysis. The mean patient age was 57.3 ± 12.5 years, and 54.3% of the patients were women. The most common diagnoses were breast cancer (*n* = 23, 25.1%), lung cancer (*n* = 21, 22.9%), gastrointestinal cancer (*n* = 20, 20.9%), and lymphoma (*n* = 16, 17.7%). A hemoglobin response was observed in 36 (39.1%), 53 (57.6%), and 61 (66.3%) patients in the third, fifth, and eighth weeks, respectively. The mean increase in hemoglobin levels from the baseline to the end of treatment was 1.77 ± 1.30 g/dL. Baseline values of hepcidin (*p =* 0.008), total iron binding capacity (*p =* 0.014), ferritin (*p =* 0.048), and CRP (*p =* 0.044) were significantly different between the responder and nonresponder groups. Multiple logistic regression analysis for baseline anemia-related biochemical variable significantly associated with the hemoglobin response showed that only baseline hepcidin level was a significant factor for hemoglobin response (odds ratio = 0.95, 95% confidence interval 0.90–1.0, *p =* 0.045). Hemoglobin responders had significantly lower hepcidin levels than nonresponders (mean [±standard deviation], 13.45 [±14.71] versus 35.22 [±40.470 ng/ml]; *p =* 0.007). However, our analysis had some limitations such as the different patient characteristics in the studies that were included, institutional differences in the measurement of hepcidin level, and missing data on long-term safety. Therefore, our findings need further validation.

**Conclusions:**

Intravenous ferric carboxymaltose (1,000 mg) monotherapy increases hemoglobin levels without serious adverse events in patients with cancer. Hepcidin is a useful biomarker for predicting iron requirement in cancer patients.

**Trial registration:**

Clinicaltrials.gov NCT02599012

## Abstract 2

Youjin Kim et al investigate the efficacy of ferric carboxymaltose for treating anemia in patients with cancer.

## Author summary

**Why was this study done?:**

- Anemia is a common complication in cancer patients undergoing treatment, and it has markedly negative effects on the patient’s quality of life and overall prognosis.
- Given the potential risks related to the use of blood transfusions and erythropoiesis-stimulating agents (ESAs), and considering the growing knowledge on iron pathophysiology and its implications in cancer- and chemotherapy-induced anemia (CIA), intravenous (IV) iron administration provides a promising therapeutic approach.
- However, there is a lack of literature on definitive guidelines for iron therapy.

**What did the researchers do and find?:**

- This was a prospective single-arm Phase II clinical trial wherein we enrolled 103 patients with solid cancers and evaluated them over an 8-week observation period.
- We demonstrated that IV ferric carboxymaltose safely improved the hemoglobin levels without ESAs in patients undergoing cancer treatment.
- Furthermore, baseline hepcidin level was found to be a predictive biomarker for the need for iron supplementation in patients with CIA.

**What do these findings mean?:**

- These findings provide evidence for establishing the usefulness of IV iron treatment for CIA. Moreover, we determined that hepcidin level might be a more reliable marker than ferritin and transferrin saturation levels for detecting functional and absolute iron deficiencies in patients with CIA.
- However, our analysis had some limitations, such as the different patient characteristics in the studies included in our work, institutional differences in the measurement of hepcidin level, and missing data on long-term safety.

## Introduction

Anemia is a common complication in patients with cancer, particularly in those receiving chemotherapy. It has markedly negative effects on the quality of life and overall prognosis [1,2]. Cancer- and chemotherapy-induced anemia (CIA) may substantially affect survival and treatment efficacy, delay or limit therapy, and contribute to both fatigue and a diminished health‐related quality of life [3]. Therefore, proper management of anemia may have a positive impact on treatment outcomes and is a crucial component of cancer management [4,5]. Current therapeutic options for CIA include red blood cell transfusions, erythropoiesis-stimulating agent (ESA) therapy, and iron supplementation according to iron status [6,7], with blood transfusions being the most commonly used modality. Although blood transfusions are effective in increasing hemoglobin (Hb) levels immediately, they only yield transient benefits, and concerns regarding their negative effects have prompted clinicians to consider alternative treatment approaches [8]. Previous studies have indicated that ESAs reduced transfusion requirements in cancer patients [9,10]. However, given their association with an increased risk of venous thromboembolic events [11–13], clinicians should be cautious regarding the use of ESAs. Because of this insight, the risks associated with blood transfusions, and the growing knowledge regarding iron pathophysiology and its implication in CIA, intravenous (IV) iron administration presents a promising and potentially valuable therapeutic approach [14,15]. However, definitive guidelines for iron therapy are lacking, as relevant data remain scarce, and prospective studies are needed.

The pathogenesis of CIA is complex and typically multifactorial, with iron deficiency (ID) being a major and potentially treatable contributor. Alternatively, in patients with cancer, ID can be caused by multiple concurrent mechanisms, including bleeding (e.g., in gastrointestinal cancers or after surgery), malnutrition, medication, and hepcidin-driven iron sequestration into macrophages with subsequent iron-restricted erythropoiesis [16]. There are numerous blood-based biomarkers for assessing iron stores, but each one has limitations. The National Comprehensive Cancer Network (NCCN) guidelines define absolute ID or functional ID using transferrin saturation (TSAT) and ferritin as iron status variables [17]. However, CIA closely resembles anemia of chronic disease (ACD), with patients frequently exhibiting up-regulated serum ferritin levels, even in iron-deficient states [16]. The falsely elevated ferritin levels in patients with cancer may mask their true iron levels and thus prevent them from receiving appropriate treatment. Therefore, a new real-time biomarker is still needed to identify patients requiring iron supplementation.

The present study aimed to investigate the efficacy of IV ferric carboxymaltose (FCM) monotherapy for the treatment of CIA and to identify predictive biomarkers for iron therapy in patients with cancer.

## Methods

### Study design

This was a prospective single-arm Phase II clinical trial conducted at a single institute (Samsung Medical Center, South Korea), from September 2016 to September 2017, over an 8-week observation period. Data were collected from patient medical records and transferred to a secure web-based electronic case report form by physicians or trained study nurses. The cutoff date for data collection was February 2018.

The primary end point was the proportion of patients achieving an Hb response at any point (week 3, 6, or 8) after a Ferinject infusion. The secondary end points were (1) the mean change in Hb level from the baseline to week 8; (2) changes in anemia-related biochemical variables from the baseline to weeks 3, 6, and 8; and (3) predictive exploratory biomarkers of an Hb response. The protocol was reviewed and approved by the institutional review board of the Samsung Medical Center (IRB no. 2015-01-011), and the trial was conducted in accordance with the Declaration of Helsinki. This trial was registered at www.clinicaltrials.gov (registration no.: NCT02599012), and written informed consent was obtained from all patients prior to participation. After approval from www.clinicaltrials.gov, we initiated recruitment on September 21, 2016. The date of the first Ferinject administration was September 23, 2016.

### Patient characteristics

The eligibility criteria were (1) age ≥ 18 years; (2) diagnosis of solid malignancy or lymphoma, irrespective of tumor type; (3) receipt of chemotherapy, including therapy with targeted agents, for at least 8 weeks prior to study enrollment or scheduled receipt of chemotherapy during the trial; (4) baseline Hb levels of 8.0–10.5 g/dL or a decrease of >2 g/dL in Hb levels during chemotherapy; (5) Eastern Cooperative Oncology Group performance status 2; and (6) life expectancy of at least 6 weeks. The exclusion criteria were (1) receipt of oral or IV iron supplements or ESAs up to 4 weeks before inclusion; (2) an active infection; (3) neoplastic bone marrow infiltration; (4) ferritin > 800 ng/ml and TSAT ≥ 50%; and (5) ongoing bleeding.

### Treatment plan and assessment

Patients received an IV FCM infusion on the first day (visit 1) of chemotherapy or targeted therapy. Ferinject (1,000 mg iron) was diluted in 250 ml of sterile 0.9% sodium chloride solution and administered as a single infusion over at least 15 min. Blood tests were performed at baseline (visit 1) and again in the third week (visit 2), sixth week (visit 3), and eighth week (visit 4). Total iron binding capacity (TIBC), TSAT, iron, ferritin, soluble transferrin receptor (sTfR), hepcidin, erythropoietin, interleukin-6 (IL-6), and C-reactive protein (CRP) levels were evaluated as biochemical variables of anemia. All blood samples were sent to a central laboratory (Seoul Clinical Laboratory) for analysis. Serum hepcidin levels were determined using a Hepcidin-25 bioactive high-sensitivity enzyme-linked immunosorbent assay (ELISA, DRG Instruments GmbH, Marburg, Germany) and a SpectraMax190 (Molecular Devices, Shanghai, China) microplate reader. The normal hepcidin levels reported for adults range from 0.25 to 47.66 ng/ml (2.5th–97.5th percentile [ng/ml]: 1.49–41.46), and the mean hepcidin value was 16.45 ng/Mr. The sTfR-F index was calculated based on the ratio: sTfR/log_10_ ferritin.

Treatment-related adverse events (AEs) were recorded at each visit. The severities of AEs were evaluated according to the National Cancer Institute’s Common Terminology Criteria for Adverse Events (CTCAE, version 4.03). Patients who showed an adequate response—i.e., an increase of at least 1 g/dL or more in Hb levels or an Hb correction ≥ 11.0 g/dL (only if the baseline Hb level was 8.0–10.5 g/dL)—after the start of IV iron treatment without a transfusion or ESAs were categorized as “responders.” Those who had a <1 g/dL increase in Hb level were classified as “nonresponders.” The efficacy of FCM was analyzed based on the proportion of responders. Patients with complete Hb data were included in the final analysis.

### Statistical analysis

The Hb response rate of interest (Ha) was 30%, and the rate of no interest (Ho) was 15%. A 2-stage design was used to calculate a total sample size of 99 assessable patients: 42 in stage 1 and 57 in stage 2, based on α and β of 0.05 (power 95%). If 7 or fewer patients achieved Hb response in stage 1, the study was to be terminated. If ≥8 out of 42 patients demonstrated Hb response in stage 1, an additional 57 assessable patients were to be enrolled in stage 2. The number of patients in the second stage of the trial was based on an adaptation of the multistage designs by Jung and colleagues [18], and the true Hb response rate was estimated according to a previous study by Jung and Kim [19]. The intention-to-treat (ITT) population comprised patients who received at least 1 dose of the study drug, whereas the per-protocol (PP) population comprised patients without any protocol violations and for whom all predictive factors were assessed on schedule and recorded. Missing data were not imputed. The PP population was used for efficacy analysis, and the ITT population was used for safety analysis. Safety was assessed by summarizing the frequency and grade of adverse reactions. Two-sample *t* test was applied to analyze the mean differences between responders and nonresponders. The 2-sided level of significance was set at 0.05. Logistic regression was used to evaluate whether Hb response was affected by baseline levels of hepcidin, iron indices, and levels of IL-6, CRP, and erythropoietin. Continuous data were expressed as mean ± standard deviation (SD). All statistical analyses were performed using the Statistical Package from SAS version 9.4 (SAS Institute, Cary, NC).

## Results

### Baseline patient characteristics

**Fig 1:**
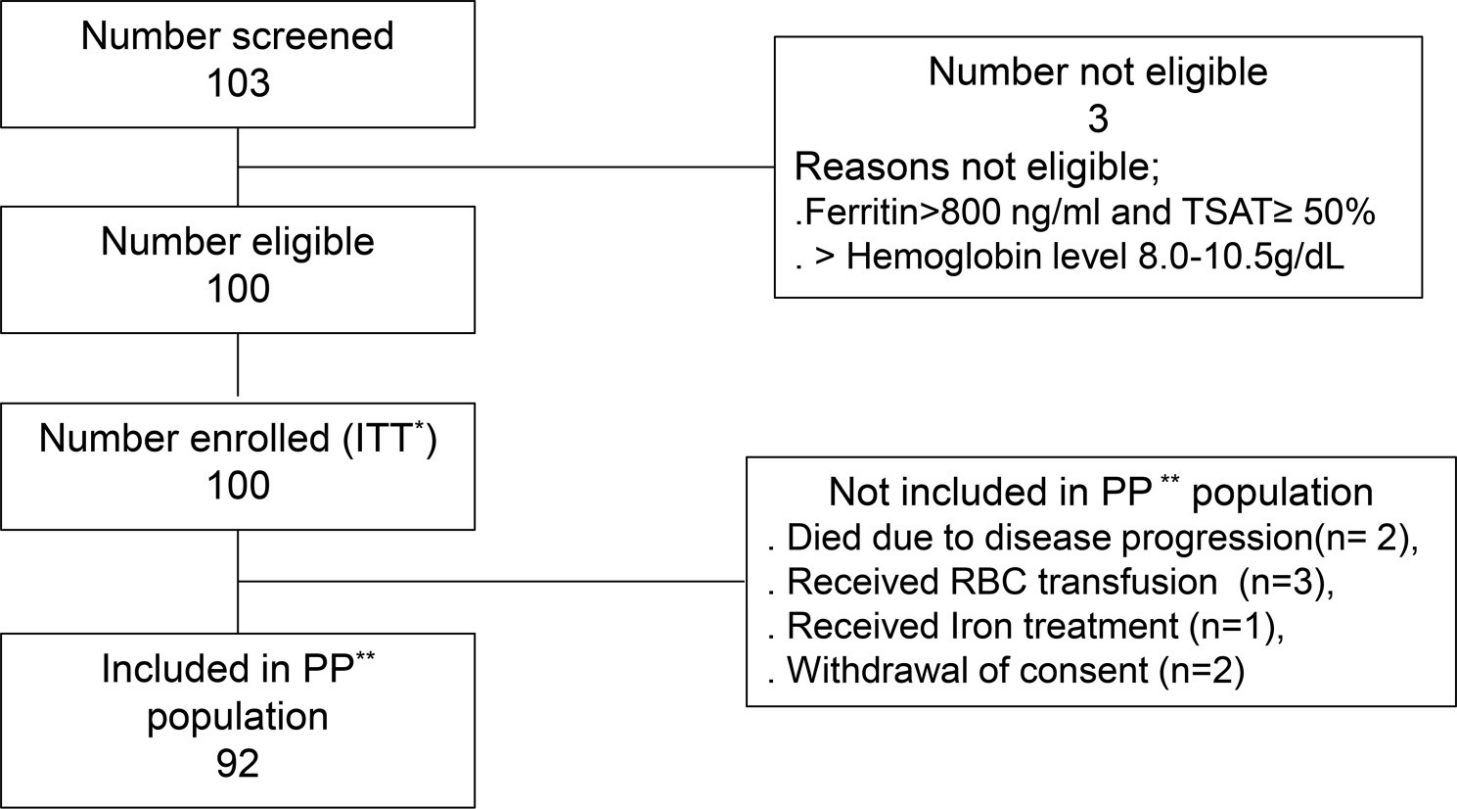
Patient screening and enrollment. ITT*, intention-to-treat; PP**, per-protocol; RBC, red blood cell; TSAT, transferrin saturation.

Among the 103 patients screened, 3 patients were excluded because their Hb or iron profile (ferritin > 800 ng/ml and TSAT ≥ 50%) did not meet the inclusion criteria set for FCM infusion. Enrollment proceeded to stage 2 because 17/43 patients achieved an Hb response during stage 1. Thus, 92 patients were included in the PP population. The patient enrollment flowchart is shown in Fig 1, and the baseline characteristics of the PP population are detailed in Table 1. The mean patient age was 57.3 ± 12.5 years, and 54.3% of the patients were women. Patients were categorized according to the treatment objective: neoadjuvant, 5 patients; adjuvant, 22; definitive, 16; and palliative, 49. Based on treatment modality, 87 patients were treated with chemotherapy alone or chemoradiotherapy, whereas 5 were treated with targeted chemotherapy. At baseline, the Hb, serum ferritin, and TSAT levels were 9.30 (±0.58) g/dL, 359.1 (±587.8) mg/L, and 17.45% (±9.89%), respectively.

**Table 1:** Patient characteristics (per-protocol population).

Clinical characteristics		*n* (%)	
Sex, *n* (%)	Male	42	(45.7)
	Female	50	(54.3)
Age (years), mean ± SD		57.32	(12.7)
Cancer type, *n* (%)	Breast cancer	23	(25.1)
	NSCLC	21	(22.9)
	Lymphoma	16	(17.7)
	Gastric cancer	12	(12.1)
	Colorectal cancer	8	(8.8)
	Sarcoma	4	(4.3)
	SCLC	3	(3.4)
	Hepatobiliary cancer	2	(2.3)
	Others^a^	3	(3.4)
Treatment purpose, *n* (%)	Adjuvant	22	(23.9)
	Definitive	16	(17.4)
	Neoadjuvant	5	(5.4)
	Palliative	49	(53.3)
Treatment modality, *n* (%)	Chemotherapy alone	83	(90.3)
	Chemoradiotherapy	4	(4.3)
	Targeted agent	4	(4.3)
	Targeted agent + radiotherapy	1	(1.1)
Status of treatment, *n* (%)	Ongoing	77	(83.7)
	Stopped	15	(16.3)
Previous anemia	Yes	8	(8.7)
treatment, *n* (%)	No	84	(91.3)
Anemia treatment type, *n* (%)	ESAs	3	(3.4)
	Transfusion^b^	6	(6.5)

One patient was treated with ESA and transfusion.

^a^Others; germinoma of the brain, testicular germ cell tumor, and esophageal cancer.

^b^Transfusion: 2 units RBCs, 4 patients; 4 units RBCs, 2 patients.

Abbreviations: ESA, erythropoietin-stimulating agents; NSCLC, non-small cell lung cancer; RBC, red blood cell; SCLC, small cell lung cancer; SD, standard deviation.

### Hb response rate according to TSAT and ferritin levels

The effects of IV iron administration and changes in Hb levels from the baseline to each visit are summarized in Table 2. There were 92 patients who were eligible for the efficacy analysis. Among them, 61 (66.3%) achieved an Hb response. Specifically, 36 (39.1%), 56 (60.9%), and 61 (66.3%) responders were observed at weeks 3, 6, and 8, respectively. In the PP population, the mean increase in Hb levels from baseline to the end of treatment was 1.77 ± 1.30 g/dL. Interestingly, among those without absolute ID anemia (IDA, defined as ferritin levels < 30 ng/ml or TSAT < 20%), the Hb response rate was 58.3% (42/72 patients). Furthermore, of the 72 patients without absolute IDA, 56 had serum ferritin levels of 30–500 ng/ml, 6 had serum ferritin levels of 500–800 ng/ml and TSAT < 50%, and 10 had ferritin levels > 800 ng/ml or TSAT ≥ 50%. The corresponding Hb response rates in these populations were 60.7% (34/56), 50% (3/6), and 50% (5/10), respectively (S1 Table).

**Table 2:** Hb level at each visit and mean change from baseline.

		Third week	Sixth week	Eighth week
Hb response, *n* (%)	Responders^a^	36 (39.1)	53 (57.6)	61 (66.3)
	Nonresponders	56 (60.9)	39 (42.4)	31 (33.7)
Hb level (mean ± SD)		9.85 ± 1.31	10.72 ± 1.26	11.15 ± 1.35
Hb change^b^ (mean ± SD)		0.55 ± 1.16	1.35 ± 1.17	1.77 ± 1.30

^a^Responders were patients that fulfilled the following criteria after ferric carboxymaltose injection

1. Patients with a ≥1.0 g/dL increase in Hb levels compared with the baseline Hb level.

2. Patients with Hb levels > 11.0 g/dL.

^b^Change from baseline.

Abbreviations: Hb, hemoglobin; SD, standard deviation.

### Anemia-related biochemical variables

The levels of hepcidin, IL-6, sTfR, TSAT, TIBC, CRP, erythropoietin, and ferritin at the baseline were compared between responders and nonresponders (Table 3). Baseline hepcidin levels were significantly lower in responders than in nonresponders (13.45 [±14.71] versus 35.22 [±40.47] ng/ml, *p =* 0.008). Notably, among the 6 patients who had serum ferritin levels of 500–800 ng/ml and TSAT < 50%, the mean baseline hepcidin levels were also different between responders (*n =* 3) and nonresponders (*n =* 3) (8.71 [±2.38] versus 36.29 [±17.98], *p =* 0.110; S2 Table). Baseline TIBC, CRP, ferritin levels, and sTfR-F index showed statistically significant differences between responders and nonresponders. Meanwhile, the baseline TSAT (%) did not differ significantly between responders and nonresponders. The mean changes in anemia-related biochemical variables from baseline to visit 4 have been provided in S5 Table. Multiple logistic regression analysis for baseline factors significantly associated with an Hb response showed that only the baseline hepcidin level was a significant factor for Hb response (odds ratio [OR] = 0.96, 95% confidence interval [CI] 0.92–1.00, *p =* 0.037; Table 4).

**Table 3:** Anemia-related biochemical variables at baseline.

Variable		Total	Responder^a^	Nonresponder	*p*-Value
Hepcidin (ng/ml)	*n*	86	56	30	0.008
	Mean ± SD	21.04 ± 28.42	13.45 ± 14.71	35.22 ± 40.47	
IL-6 (pg/ml)	*n*	81	52	29	0.189
	Mean ± SD	19.00 ± 37.00	14.34 ± 28.70	27.35 ± 47.95	
sTfR (μg/ml)	*n*	92	61	31	0.377
	Mean ± SD	1.46 ± 0.60	1.50 ± 0.65	1.38 ± 0.47	
Iron (μg/ml)	*n*	92	61	31	0.678
	Mean ± SD	52.00 ± 3.53	52.82 ± 34.40	5.39 ± 21.38	
TSAT (%)	*n*	92	61	31	0.875
	Mean ± SD	17.45 ± 9.89	17.34 ± 11.09	17.65 ± 7.11	
TIBC (μg/dL)	*n*	92	61	31	0.014
	Mean ± SD	306.48 ± 73.45	32.84 ± 77.19	278.23 ± 56.58	
CRP (mg/dL)	*n*	92	61	31	0.044
	Mean ± SD	1.28 ± 3.11	.65 ± 1.17	2.52 ± 4.92	
Erythropoietin (mIU/ml)	*n*	92	61	31	0.152
	Mean ± SD	59.67 ± 6.45	52.30 ± 5.38	74.16 ± 75.37	
Ferritin (ng/ml)	*n*	92	61	31	0.049
	Mean ± SD	359.1 ± 587.8	249.2 ± 357.8	575.3 ± 848.1	
WBC (10^3^/μl)	*n*	92	61	31	0.429
	Mean ± SD	5.64 ± 3.06	5.44 ± 2.68	6.04 ± 3.70	
PLT (10^3^/μl)	*n*	92	61	31	0.822
	Mean ± SD	254.6 ± 108.1	252.8 ± 101.3	258.2 ± 121.9	
ANC (10^3^/μl)	*n*	92	61	31	0.329
	Mean ± SD	3.57 ± 2.62	3.38 ± 2.47	3.95 ± 2.91	
Reticulocyte (%)	*n*	90	59	31	0.460
	Mean ± SD	2.83 ± 1.34	2.75 ± 1.44	2.97 ± 1.14	
Corrected reticulocyte count (%)	*n*	90	59	31	0.564
	Mean ± SD	1.94 ± 0.90	1.89 ± 0.97	2.01 ± 0.78	
sTfR-F index	*n*	92	61	31	0.003
	Mean ± SD	0.89 ± .0.84	1.03 ± 0.97	0.60 ± .0.36	

^a^Responders were patients that fulfilled the following criteria after ferric carboxymaltose injection

1. Patients with a ≥1.0 g/dL increase in Hb levels from the baseline level.

2. Patients with Hb levels > 11.0 g/dL.

Abbreviations: ANC, absolute neutrophil count; CRP, C-reactive protein; Hb, hemoglobin; IL-6, interleukin-6; PLT, platelet count; SD, standard deviation; sTfR, soluble transferrin receptor; sTfR-F index, sTfR/log_10_ (ferritin); TIBC, total iron binding capacity; TSAT, transferrin saturation; WBC, white blood cell.

**Table 4:** Odds ratios and 95% confidence intervals for hemoglobin response.

Variables	Reference	Odds ratio	95% Confidence interval	*p*-Value
			(Lower, upper)	
Sex	1 = Female	0.62	0.18–2.21	0.464
	0 = Male			
Age	1 year	1.04	0.99–1.09	0.162
Previous anemia treatment	1 = Yes	1.80	0.23–13.77	0.573
	0 = No			
Status of treatment	1 = Ongoing	0.82	0.16–4.35	0.819
	0 = Stopped			
Hemoglobin	1 g/dL	1.05	0.35–3.14	0.929
Hepcidin-25	1 ng/ml	0.96	0.92–1.00	0.037
IL-6	1 pg/ml	1.00	0.98–1.03	0.788
Iron	1 μg/dL	0.98	0.94–1.03	0.487
TSAT	1%	1.06	0.91–1.24	0.430
UIBC	1 μg/dL	1.00	0.97–1.03	0.99
CRP	1 mg/dL	0.85	0.38–1.92	0.743
Erythropoietin	1 mIU/ml	0.99	0.98–1.01	0.187
White blood cell	1 × 10^3^/μl	1.82	0.75–4.46	0.188
Platelet	1 × 10^3^/μl	1.00	1.00–1.01	0.817
Absolute neutrophil count	1 × 10^3^/μl	0.53	0.19–1.47	0.223
Reticulocyte count	1%	0.80	0.52–1.24	0.318
sTfR-F index	1	4.34	0.56–33.77	0.161

Dependent variable: Hemoglobin response (probability modeled is “Response”).

Abbreviations: CRP, C-reactive protein; IL-6, interleukin-6; sTfR, soluble transferrin receptor; sTfR-F index, sTfR/log_10_(ferritin); TSAT, transferrin saturation; UIBC, unsaturated iron binding capacity.

### Hb response rate according to hepcidin levels

**Fig 2:**
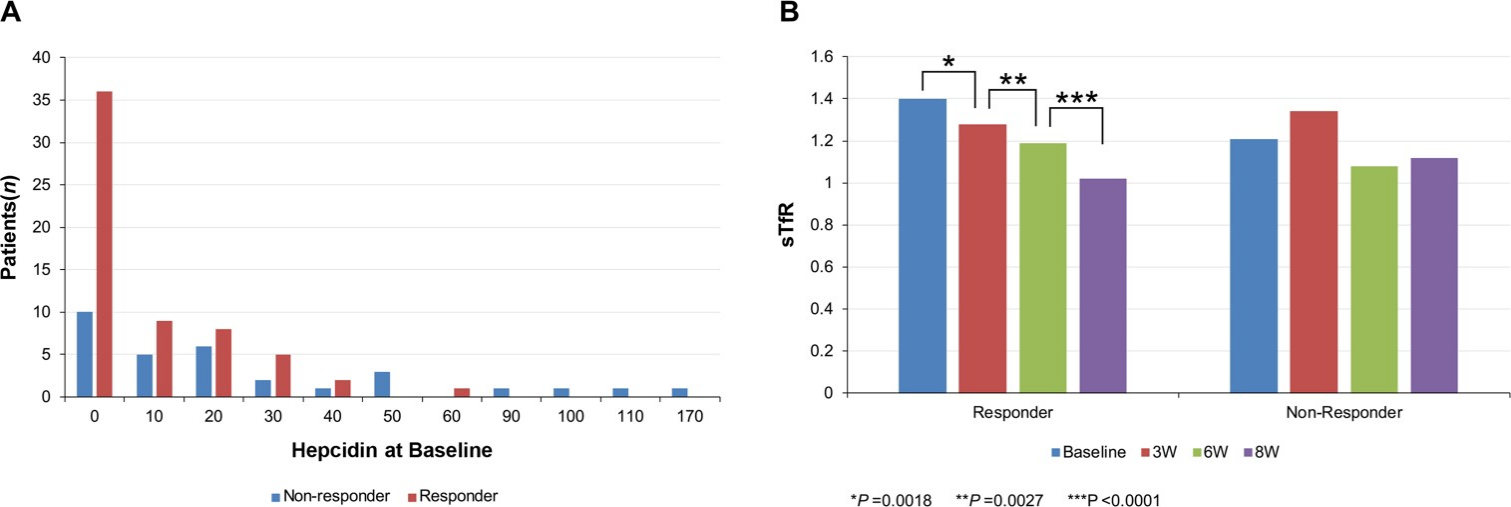
Hemoglobin response according to baseline hepcidin levels and the changes in mean sTfR. (A) Number of patients who achieved hemoglobin response according to baseline hepcidin levels. (B) Analysis of the changes in mean sTfR levels of responders and nonresponders from baseline to 8W. sTfR, soluble transferrin receptor; W, weeks.

The number of responders based on hepcidin levels is shown in Fig 2A. Furthermore, baseline hepcidin was analyzed both as a continuous variable and divided into 4 percentiles (50th, 80th, 90th, and 95th), and hepcidin levels were compared with the Hb response rates to characterize responders (S3 Table). Using a normal laboratory reference value of 16.45 ng/ml, there were 43/61 responders and 14/31 nonresponders (*p =* 0.023, 95% CI 0.011–0.055). The 50th percentile baseline hepcidin level for “responders” was 13.45 ± 14.71 ng/ml, whereas the 80th, 90th, and 95th percentiles were 25.96, 30.55, and 34.11 ng/ml, respectively. Using a reference value of 25.96 ng/ml, there were 49/61 responders and 14/31 nonresponders (*p =* 0.018, 95% CI 0.005–0.048). A significant number of patients achieved an Hb response when the 90th percentile cutoff value of 30.55 ng/ml was used (55/61 responders and 21/31 nonresponders; *p =* 0.007, 95% CI 0.001–0.045). A significantly greater number of patients achieved an Hb response when the 95th percentile cutoff value of <34.11 ng/ml was used (58/61 responders and 22/31 nonresponders, *p* < 0.001).

### sTfR level at each visit

We also evaluated the Hb response ratio according to sTfR levels at baseline. The normal reference laboratory value was given as 1.0, whereas the mean value (50th percentile) of responders and the cutoff values of the responders at the 80th, 90th, and 95th percentiles were 1.40, 1.93, 2.23, and 2.69, respectively. The percentage of responders was not significantly associated with any of the cutoff sTfR values (all *p* > 0.05; S4 Table). The number of responders according to sTfR levels is detailed in S1 Table. Analysis of the changes in the mean sTfR levels of responders and nonresponders from the baseline to 8 weeks showed that mean sTfR values decreased significantly from the baseline to the third week, from the third week to the sixth week, and from the sixth week to the eighth week, only in responders (Fig 2B). The number of responders based on sTfR levels at baseline is shown in S1 Fig.

### Safety

Except for 3 patients in whom screening failures occurred, 100 patients were included in the ITT population for safety analysis. Treatment-related AEs developed in 2 patients, but these AEs were mild (CTCAE grade 1) and did not necessitate discontinuation of treatment. These AEs included chest or back pain, nausea, and flushing, which subsequently resolved completely without sequelae.

## Discussion

To our knowledge, this is the first report documenting the efficacy of FCM and the association of hepcidin with treatment of patients with CIA. In the current study, we demonstrated that IV Ferinject allows for the administration of a large replenishment dose (1,000 mg of iron) to increase Hb levels safely without ESAs in patients being treated for cancer. Furthermore, baseline hepcidin levels were found to be a predictive biomarker for iron supplementation in cancer patients with anemia.

Generally, 3 types of nonoral iron substances—namely, iron dextran, ferric gluconate, and iron sucrose—have been used [20–22]. We demonstrated that FCM alone allows for administration of a high dose over a short infusion period (15–30 minutes), with longer injection interval, and without serious AEs in cancer patients. However, it has been shown that hyperferritinemia in hematologic malignancies has unfavorable effects on morbidity, and iron-chelation therapy should be initiated at a serum ferritin level of ≥1,000 ng/ml [23]. A long-term assessment is needed to clarify the effect of repeated FCM administration for patients with solid cancers, wherein ferritin is frequently elevated.

Currently, the NCCN guidelines recommend iron supplementation, especially IV iron supplementation, for both absolute ID (ferritin < 30 ng/ml and TSAT < 20%) and functional ID in patients receiving ESAs (ferritin 30–500 ng/ml and TSAT < 50%) [1]. However, in patients with cancer, ferritin is frequently up-regulated even in an iron-deficient state, which can mask the true iron levels [7,16]. Furthermore, although blood transfusions are the most commonly used treatment modality for anemia in cancer patients, it can result in increased plasma iron levels [24], hindering the evaluation of iron metabolism in patients receiving transfusions. In potential patients with functional ID (ferritin > 500–800 ng/ml and TSAT < 50%), IV iron therapy is not required, or it is only considered for some patients. Patients with ferritin levels > 800 ng/ml or TSAT ≥ 50% do not require IV iron therapy according to the current NCCN guidelines. However, some patients in both these groups exhibited an Hb response to IV iron therapy in the current study. The inaccuracy of ferritin levels as a marker for anemia in cancer patients shows that a new biomarker is needed as a reference for iron therapy in CIA.

The measurement of serum hepcidin is a promising method for assessing ID status [25]. We demonstrated that Hb responders had significantly lower hepcidin levels than Hb nonresponders to IV iron monotherapy without ESAs. Similar results have been reported in a small sample of patients with CIA [26]. Iron homeostasis is regulated in part by hepcidin, whose expression is affected differently by inflammation and ID [27,28]. Tumors induced inflammatory reactions that increase the levels of inflammatory cytokines such as IL‐6. Upon binding to its receptor, IL‐6 initiates signaling through activated Janus kinase (JAK) 1/2 proteins to phosphorylate the activated signal transducers and activators of transcription 3 (STAT3). Next, STAT3 binds to a STAT3-responsive element on the proximal hepcidin promoter [29,30]. Long-term hepcidin production, because of its ability to inhibit ferroportin function in the duodenal enterocytes and reticuloendothelial macrophages, leads to poor iron absorption in the gut and increased iron retention, which is a hallmark of ACD [24,31,32]. The central role played by hepcidin in the pathogenesis of ACD suggests that measurement of hepcidin levels might be a useful diagnostic tool in the evaluation of possible ACD. Conversely, limited circulating iron levels down-regulate hepcidin synthesis, allowing an influx of bioavailable iron from the duodenal enterocytes and tissue iron stores. Erythropoietic stimulators [33] and hypoxia [34] also negatively regulate hepcidin, increasing iron availability for erythropoiesis [35]. In previous studies, although ESA administration was effective for managing cancer-induced anemia, the hematologic response induced by the treatment was limited (30%–75% of treated patients) [36–40]. In many nonresponders, persistent anemia may result from functional ID that occurs with the rapid initiation of erythropoiesis stimulated by ESAs [21]. This has also been shown in a study on patients with CIA who were treated with darbepoetin. Steensma and colleagues showed that patients with serum hepcidin levels in the lower 2 tertiles (>20.2–64.3 ng/ml) experienced a better clinical response to ESA and high doses of IV iron than did patients with lower hepcidin levels who did not receive a high dose of IV iron [21,40]. Based on these results, patients with lower levels of hepcidin were more likely to benefit from intensive iron supplementation, suggesting that hepcidin level is a reliable biomarker for detecting functional ID.

Furthermore, hepcidin-related inhibition of iron absorption in the gut explains why there is little or no response to oral iron supplementation. IV administration of iron may play a role in overcoming resistance to hepcidin-related reduction in iron availability for erythroblasts and ultimately lead to the correction of anemia in these patients. Therefore, predictive indicators for IV iron therapy are important for treating CIA. In this study, we found that there were more responders to FCM in the group of patients with serum hepcidin levels in the lower 97.5th percentile (≤34.11 ng/ml) than among those with serum levels in other percentiles, according to a Hepcidin-25 bioactive high-sensitivity ELISA. However, because of the highly variable cutoff reported with different assays, standardization of hepcidin assays is necessary before making practical recommendations. These findings have important implications in showing that hepcidin is more accurate than TSAT and ferritin in identifying concomitant ID in patients with inflammation, such as in patients with cancer or autoimmune disorders. The measurement of hepcidin may be a useful addition to the differential diagnosis of tumor-related ACD and IDA, thereby guiding corrective treatment.

Interestingly, we observed that the mean sTfR levels decreased significantly only in responders with every visit, whereas the baseline sTfR values were not significantly different between responders and nonresponders. Plasma sTfR concentration reflects the receptor density on the cells and the number of cells expressing receptors. Therefore, it is closely related to cellular iron demands and to the erythroid proliferation rate [41]. In recent years, sTfR has been used as a sensitive, early, and highly quantitative novel marker for iron depletion, increasing in proportion to tissue iron deficits [42]. Our results showed that the mean sTfR value decreased significantly at each visit following IV iron therapy only in responders, whereas no significant changes were observed in the nonresponders. The sTfR is a marker for the activity and size of the erythrocyte precursors in the bone marrow and is not directly influenced by inflammation [43]. In the absence of data for identifying significant predictors of response to IV iron therapy in patients with CIA, our results support the use of sTfR levels, but not baseline sTfR levels, in monitoring the erythropoietic response to IV iron supplementation. In particular, this will allow early prediction of the response before changes in Hb become apparent.

Several limitations of our analysis should be duly considered. First, the type of patients, malignancies, and chemotherapy regimens were heterogeneous. Second, considering the institutional differences in the measurement of hepcidin, further standardization and investigation is probably required before hepcidin levels are recommended for routine and widespread clinical use. Third, given the characteristics of cancers that may progress over time and need for continued treatment, we require more patients and a longer follow-up time to assess the appropriate duration of Ferinject treatment. Fourth, the long-term safety of repeated IV iron administration should be further examined in prospective trials. To our knowledge, this is the first prospective Phase II clinical trial to assess the efficacy and safety of IV FCM administration as a monotherapy in CIA patients. Furthermore, we simultaneously performed an exploratory analysis of representative iron status variables including hepcidin.

In conclusion, our results suggest that FCM alone can improve the Hb response without serious AEs in patients with CIA. Hepcidin may be a more reliable marker than ferritin and TSAT for detecting functional ID as well as absolute ID in patients with cancer. Moreover, sTfR levels are helpful for monitoring erythropoietic responses to IV iron supplementation. Further trials are warranted to investigate the clinical value of IV iron in CIA, identify patients who will benefit from iron therapy, and confirm the potential role of hepcidin as a predictive marker of functional ID.

## Supporting information

S1 Consort Checklist (DOC)

S1 Table Classification of responders according to ferritin levels and TSAT. TSAT, transferrin saturation.
(DOCX)

S2 Table Anemia-related biochemical variables at baseline in patients with ferritin levels of 500–800 ng/ml and TSAT < 50%. TSAT, transferrin saturation.
(DOCX)

S3 Table Hemoglobin response rates according to baseline hepcidin levels. (DOCX)

S4 Table Hemoglobin response rates according to baseline sTfR levels. sTfR, soluble transferrin receptor.
(DOCX)

S5 Table Anemia-related biochemical variables mean change from baseline to visit 4. (DOCX)

S1 Fig Hemoglobin response status according to sTfR levels at baseline. sTfR, soluble transferrin receptor.
(TIF)

## Abbreviations

ACD: anemia of chronic disease
AE: adverse event
CI: confidence interval
CIA: chemotherapy-induced anemia
CRP: C-reactive protein
CTCAE: Common Terminology Criteria for Adverse Events
ELISA: enzyme-linked immunosorbent assay
ESA: erythropoiesis-stimulating agent
FCM: ferric carboxymaltose
Ha: Hb response rate of interest
Hb: hemoglobin
Ho: rate of no interest
ID: iron deficiency
IDA: ID anemia
IL-6: interleukin-6
ITT: intention-to-treat
IV: intravenous
JAK: Janus kinase
NCCN: National Comprehensive Cancer Network
OR: odds ratio
PLT: platelet count
PP: per-protocol
SD: standard deviation
STAT3: signal transducers and activators of transcription 3
sTfR: soluble transferrin receptor
TIBC: total iron binding capacity
TSAT: transferrin saturation
UIBC: unsaturated iron binding capacity
