# Supplementary material for: Efficacy of intravenous iron treatment for chemotherapy-induced anemia: A prospective Phase II pilot clinical trial in South Korea
Source: PLoS Med. 2020 Jun 8;17(6):e1003091. doi: 10.1371/journal.pmed.1003091 (PMC7279571; doi:10.1371/journal.pmed.1003091)
Supplement: S1 Table — TSAT, transferrin saturation. (DOCX) [file pmed.1003091.s002.docx]

**S1 Table. Classification of responders according to ferritin levels and TSAT**

|  | Iron status | Ferritin >30–500 ng/ml  and TSAT <50% | Ferritin >500–800 ng/ml  and TSAT <50% | Ferritin >800 ng/ml  or TSAT ≥50% |
| --- | --- | --- | --- | --- |
| Total, n (%) |  | 56 | 6 | 10 |
| Hb response (≥1) | ^a^Responder | 34 (60.7) | 3 (50.0) | 5 (50.0) |
|  | Non-responder | 22 (39.3) | 3 (50.0) | 5 (50.0) |
| Hb response (≥1.5) | ^a^Responder | 24 (42.9) | 3 (50.0) | 3 (30.0) |
|  | Non-responder | 32 (57.1) | 3 (50.0) | 7 (70.0) |
| Hb response (≥2) | ^a^Responder | 19 (33.9) | 3 (50.0) | 2 (20.0) |
|  | Non-responder | 37 (66.1) | 3 (50.0) | 8 (80.0) |

**Abbreviations:** Hb, hemoglobin; TSAT, transferrin saturation.

^a^ Responders were defined as the following after ferric carboxymaltose injection:

i) Patients with ≥1.0 g/dl increase in Hb levels over the baseline Hb level.

ii) Patients with Hb levels >11.0 g/dl.
