# Supplementary material for: Efficacy of intravenous iron treatment for chemotherapy-induced anemia: A prospective Phase II pilot clinical trial in South Korea
Source: PLoS Med. 2020 Jun 8;17(6):e1003091. doi: 10.1371/journal.pmed.1003091 (PMC7279571; doi:10.1371/journal.pmed.1003091)
Supplement: S2 Table — TSAT, transferrin saturation. (DOCX) [file pmed.1003091.s003.docx]

**S2 Table. Baseline anemia-related biochemical variables in patients with ferritin levels of 500**–**800 ng/ml and TSAT <50%**

| Biochemical variables | | Total | ^a^Responders | Non-responders | *p*-value |
| --- | --- | --- | --- | --- | --- |
| Hepcidin | n | 6 | 3 | 3 |  |
|  | Mean±SD | 22.50±18.97 | 8.71±2.38 | 36.29±17.98 | 0.110 |
| IL-6 | n | 6 | 3 | 3 |  |
|  | Mean±SD | 16.55±16.26 | 5.30±4.40 | 27.80±16.18 | 0.081 |
| sTfR | n | 6 | 3 | 3 |  |
|  | Mean±SD | 1.29±0.37 | 1.24±0.53 | 1.33±0.23 | 0.792 |
| Erythropoietin | n | 6 | 3 | 3 |  |
|  | Mean±SD | 97.20±63.35 | 59.53±62.38 | 134.87±43.43 | 0.161 |

**Abbreviations:** IL-6, interleukin-6; SD, standard deviation; sTfR, soluble transferrin receptor; TSAT, transferrin saturation.

^a^ Responders were defined as the following after ferric carboxymaltose injection:

i) Patients with ≥1.0 g/dl increase in Hb levels over the baseline Hb level.

ii) Patients with Hb levels >11.0 g/dl.
