# Supplementary material for: Efficacy of intravenous iron treatment for chemotherapy-induced anemia: A prospective Phase II pilot clinical trial in South Korea
Source: PLoS Med. 2020 Jun 8;17(6):e1003091. doi: 10.1371/journal.pmed.1003091 (PMC7279571; doi:10.1371/journal.pmed.1003091)
Supplement: S3 Table — (DOCX) [file pmed.1003091.s004.docx]

| **S3 Table. Hemoglobin response rates according to baseline hepcidin levels** | | | | | |
| --- | --- | --- | --- | --- | --- |
| Baseline hepcidin level (ng/ml) | | Responders | Non-responders | Total | *p*-value |
| n (%) | | 61 (66.3) | 31 (33.7) | 92 |  |
| ^a^50^th^ percentile | ≤13.45 | 39 (63.9) | 14 (26.4) | 53 (57.6) | ^b^0.085 |
|  | >13.45 | 22 (36.1) | 17 (43.6) | 69 (42.4) |  |
| Normal reference laboratory values | ≤16.45 | 43 (70.5) | 14 (24.6) | 57 (62.0) | ^b^0.018 |
|  | >16.45 | 18 (51.4) | 17 (48.6) | 35 (38.0) |  |
| ^a^80^th^ percentile | ≤25.96 | 49 (73.1) | 18 (26.9) | 67 (72.8) | ^b^0.023 |
|  | >25.96 | 12 (48.0) | 13 (52.0) | 25 (27.2) |  |
| ^a^90^th^ percentile | ≤30.55 | 55 (72.4) | 21 (27.6) | 76 (82.6) | ^b^0.007 |
|  | >30.55 | 6 (37.5) | 10 (62.5) | 16 (17.4) |  |
| ^a^95^th^ percentile | ≤34.11 | 58 (72.5) | 22 (27.5) | 80 (87.0) | ^c^<0.001 |
|  | >34.11 | 3 (25.0) | 9 (75.0) | 12 (13.0) |  |

^a^ Cutoff value of responders.

^b^ Chi-square test.

^c^ Fisher’s exact test.
