# Supplementary material for: Efficacy of intravenous iron treatment for chemotherapy-induced anemia: A prospective Phase II pilot clinical trial in South Korea
Source: PLoS Med. 2020 Jun 8;17(6):e1003091. doi: 10.1371/journal.pmed.1003091 (PMC7279571; doi:10.1371/journal.pmed.1003091)
Supplement: S4 Table — sTfR, soluble transferrin receptor. (DOCX) [file pmed.1003091.s005.docx]

| **S4 Table.** **Hemoglobin response rates according to baseline sTfR levels** | | | | | |
| --- | --- | --- | --- | --- | --- |
| sTfR at baseline | | Responders | Non-responders | Total | *p*-value |
| n (%) | | 61 (66.3) | 31 (33.7) | 92 |  |
| Normal reference laboratory values | ≤1.0 | 15 (65.2) | 8 (34.8) | 23 (25.0) | ^b^0.899 |
|  | >1.0 | 46 (66.7) | 23 (33.3) | 69 (75.0) |  |
| Mean value of responders (50^th^ percentile) | ≤1.40 | 31 (63.3) | 18 (36.7) | 49 (53.3) | ^b^0.510 |
|  | >1.40 | 30 (69.8) | 13 (3.2) | 43 (46.7) |  |
| ^a^80^th^ percentile | ≤1.93 | 51 (63.8) | 29 (36.3) | 80 (87.0) | ^c^0.326 |
|  | >1.93 | 10 (83.3) | 2 (16.7) | 12 (13.0) |  |
| ^a^90^th^ percentile | ≤2.23 | 55 (64.7) | 30 (35.3) | 85 (92.4) | ^c^0.417 |
|  | >2.23 | 6 (85.7) | 1 (14.3) | 7 (7.6) |  |
| ^a^95^th^ percentile | ≤2.69 | 58 (65.9) | 30 (34.1) | 88 (95.7) | ^c^1.000 |
|  | >2.69 | 3 (75.0) | 1 (25.0) | 4 (4.3) |  |

**Abbreviation:** sTfR, soluble transferrin receptor.

^a^ Cutoff value of responders.

^b^ Chi-square test.

^c^ Fisher`s exact test
