# Supplementary material for: Efficacy of intravenous iron treatment for chemotherapy-induced anemia: A prospective Phase II pilot clinical trial in South Korea
Source: PLoS Med. 2020 Jun 8;17(6):e1003091. doi: 10.1371/journal.pmed.1003091 (PMC7279571; doi:10.1371/journal.pmed.1003091)
Supplement: S5 Table — (DOCX) [file pmed.1003091.s006.docx]

**S5 Table. Mean change in anemia-related biochemical variables from baseline to visit 4**

|  | | Total | | Responder | | Non responder | | *p* value |
| --- | --- | --- | --- | --- | --- | --- | --- | --- |
| Hepcidin (ng/ml) | *n* | 68 | | 55 | | 13 | | 0.33 |
|  | Mean±SD | 26.7 | ±23.1 | 25.4 | ±22.4 | 32.4 | ±25.8 |  |
| IL-6 (pg/ml) | *n* | 51 | | 42 | | 9 | | 0.34 |
|  | Mean±SD | 14.6 | ±44.9 | ±10.5 | ±38.6 | ±33.9 | ±66.7 |  |
| STfR (µg/ml) | N | 73 | | 60 | | 13 | | 0.23 |
|  | Mean±SD | -0.4 | ±0.6 | -±0.4 | ±0.6 | -±0.2 | ±0.4 |  |
| Iron (µg/ml) | *n* | 73 | | 60 | | 13 | | 0.05 |
|  | Mean±SD | 19.9 | ±34.2 | ±22.5 | ±36.2 | ±7.8 | ±19.6 |  |
| TSAT (%) | *n* | 73 | | 60 | | 13 | | 0.31 |
|  | Mean±SD | 9.4 | ±10.8 | ±10.0 | ±11.0 | ±6.6 | ±9.9 |  |
| TIBC (µg/dl) | *n* | 73 | | 60 | | 13 | | 0.85 |
|  | Mean±SD | -44.3 | ±60.5 | -±44.9 | ±63.3 | -±41.5 | ±47.2 |  |
| CRP (mg/dl) | *n* | 73 | | 60 | | 13 | | 0.97 |
|  | Mean±SD | 0.5 | ±2.8 | ±0.6 | ±2.9 | ±0.5 | ±2.2 |  |
| Erythropoietin | *n* | 73 | | 60 | | 13 | | 0.53 |
| (mIU/ml) | Mean±SD | -29.2 | ±45.8 | -±30.8 | ±47.4 | -±21.8 | ±38.1 |  |
| Ferritin (ng/ml) | *n* | 73 | | 60 | | 13 | | 0.27 |
|  | Mean±SD | 433.1 | ±531.0 | ±389.5 | ±472.6 | ±634.0 | ±735.1 |  |
| WBC (10^3^/µl) | *n* | 73 | | 60 | | 13 | | 0.92 |
|  | Mean±SD | 0.6 | ±3.5 | ±0.6 | ±3.5 | ±0.5 | ±3.6 |  |
| PLT (10^3^/µl) | *n* | 73 | | 60 | | 13 | | 0.73 |
|  | Mean±SD | -40.8 | ±65.9 | -±42.5 | ±58.8 | -±32.8 | ±94.6 |  |
| ANC (10^3^/µl) | *n* | 73 | | 60 | | 13 | | 0.83 |
|  | Mean±SD | 0.4 | ±3.4 | ±0.5 | ±3.5 | ±0.2 | ±3.5 |  |
| Reticulocyte | *n* | 69 | | 56 | | 13 | | 0.28 |
| (%) | Mean±SD | -0.7 | ±1.5 | -±0.8 | ±1.4 | -±0.3 | ±1.8 |  |
| Corrected reti | *n* | 70 | | 57 | | 13 | | 0.98 |
| culocyte count (%) | Mean±SD | -0.3 | ±1.0 | -±0.3 | ±1.0 | -±0.3 | ±1.2 |  |

**Abbreviations:** ANC, absolute neutrophil count; CRP, C-reactive protein; IL-6, interleukin-6; PLT, platelet count; SD, standard deviation; sTfR, soluble transferrin receptor; TIBC, total iron binding capacity; TSAT, transferrin saturation; WBC, white blood cell.

^a^ Responders were defined as the following after ferric carboxymaltose injection:

i) Patients with ≥1.0 g/dl increase in Hb levels over the baseline Hb level.

ii) Patients with Hb levels >11.0 g/dl.
