# Supplementary figures and images for: Efficacy of intravenous iron treatment for chemotherapy-induced anemia: A prospective Phase II pilot clinical trial in South Korea
Source: PLoS Med. 2020 Jun 8;17(6):e1003091. doi: 10.1371/journal.pmed.1003091 (PMC7279571; doi:10.1371/journal.pmed.1003091)

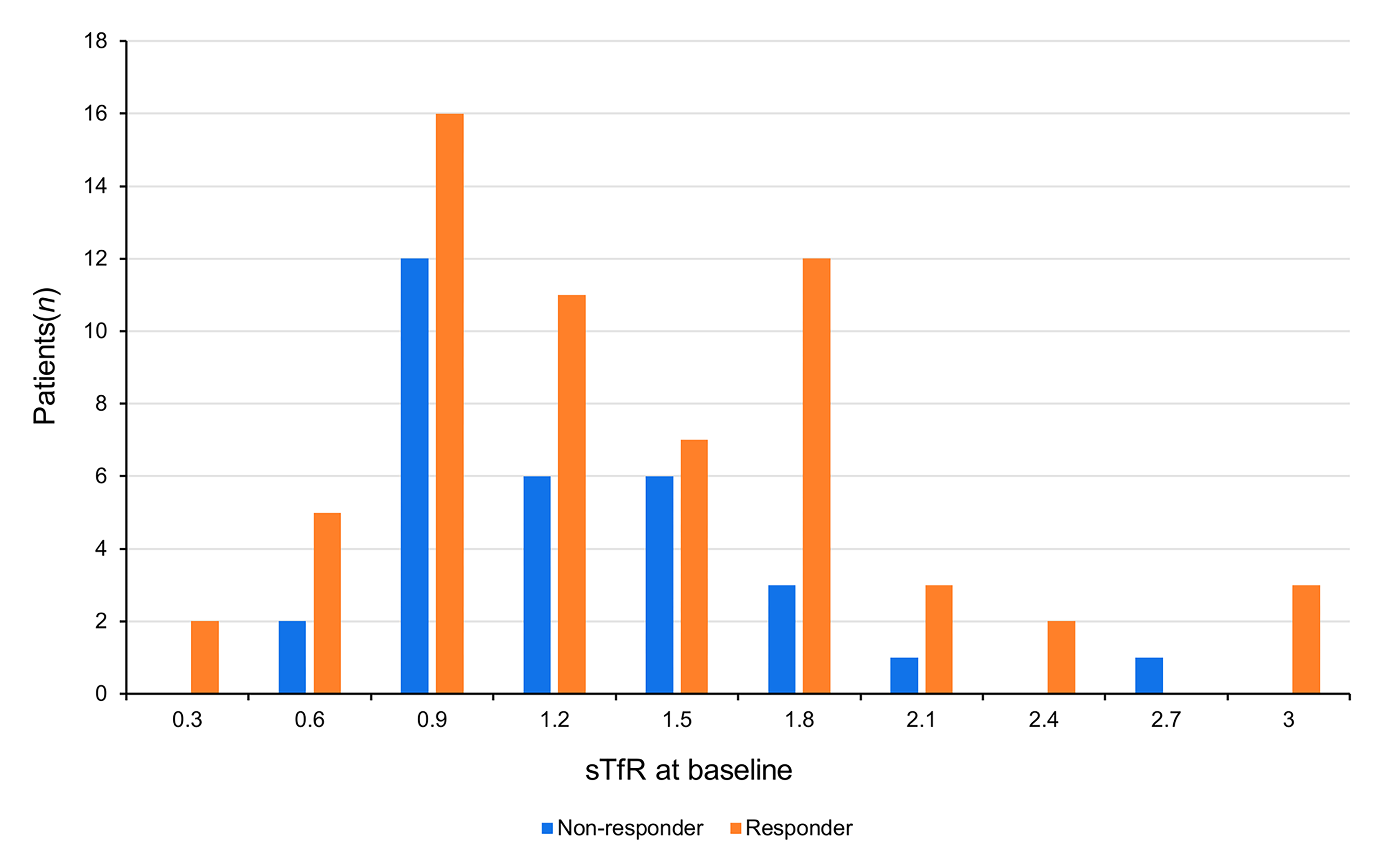

Supplement: S1 Fig — sTfR, soluble transferrin receptor. (TIF) [file pmed.1003091.s007.tif]
